# Effect of Hoagland’s nutrient solution strengths and sodium silicate on growth, yield and biochemical parameters of Carla (*Momordica Charantia* L.) under hydroponic conditions

**DOI:** 10.1038/s41598-025-92616-2

**Published:** 2025-03-06

**Authors:** Ali Majidi, Reza Shahhoseini, Hossein Salehi-Arjmand, Hamid R. Roosta

**Affiliations:** 1 Department of Medicinal Plants, Arak University, P.O.Box: 38156-8-8349, Arak, Iran; 2 Department of Horticulture, Arak University, Arak, Iran

**Keywords:** Carla, Hoagland’s solution, Sodium silicate, Yield, Environmental sciences, Plant sciences, Plant physiology, Plant reproduction

## Abstract

The medicinal species of Carla (*Momoradica charantia*) is one of the medicinal plants in Cucurbitaceae family, which has shown significant effects on the control of diabetes, blood pressure, high cholesterol and liver failure due to its valuable alkaloid and glycosidic compounds. In order to investigate the effect of different strengths of Hoagland’s nutrient solution and different levels of sodium silicate on yield components, biochemical characteristics and photosynthetic pigments of the medicinal plant Carla under hydroponic conditions, a factorial experiment in the form of a completely randomized design with three replications was carried out in the Department of Medicinal Plants of Arak University. The first factor included the concentrations of nutrient solution at four levels (¼, ½, 1 and 2 strength of Hoagland’s nutrient solution) and the second factor included sodium silicate spraying at four levels (0, 50, 100 and 150 mg L^− 1^) taken from the source of sodium silicate. The results showed that different strengths of Hoagland’s nutrient solution and different concentrations of sodium silicate had a significant effect on all growth and morphological traits. The results of the experiment showed an increase in the number of flowers and fruits in double strength Hoagland concentration compared to the control (full strength), and the lowest amounts of these traits were observed at ¼ strength Hoagland concentration. In addition, the maximum numbers of flowers and fruit, fruit diameter, fruit length, and fruit weight were obtained at concentration of 100 mg L^− 1^ sodium silicate, and the minimum values of these traits were recorded at ¼ strength Hoagland’s solution. The highest amounts of chlorophyll b and a were seen in double strength Hoagland’s solution and the lowest amounts were seen in ¼ strength Hoagland. Moreover, the amount of carotenoid was the highest in ¼ strength Hoagland and the lowest in double strength Hoagland. The maximum and minimum values of chlorophyll b and a were obtained at concentrations of 150 and 0 mg/kg sodium silicate, respectively. In general, it was found that double strength Hoagland was more effective than other concentrations on yield components and morphological parameters, and flowering and fruit harvesting times were also reduced in the mentioned treatment. On the other hand, sodium silicate at 100 and 150 mg L^− 1^ had more obvious effects on the evaluated traits and the reduction of flowering and fruit harvesting times.

## Introduction

Carla (*Momoradica charantiatia*), belonging to the Cucurbitaceae family^1^, is an edible-medicinal species that is distributed and spread all over the world^2^. Carla leaves and stems are rich in vitamins A and C, calcium, iron and phosphorus^3^. It also has phytochemical compounds such as lutein, elasterol, alkaloids, triterpenoids, polyphenols, steroids, proteins, mineral compounds and lipids^4–6^. All parts of the plant, especially roots, leaves, fruits, and seeds, have edible-medicinal uses and have a bitter taste due to the presence of momordicin. Fruit is the most important medicinal part of this plant, which is rich in vitamins A and B, iron, and insulin and quarantine polypeptides. It is also effective in reducing blood sugar^7^. In traditional medicine, it is used for the treatment of microbial infections, malaria, rheumatism, digestion and elimination of intestinal gas, stimulation of menstruation, healing of wounds, and reduction of fever, inflammation, high blood pressure, and abortion^8^. Carla leaf extract is effective in the treatment of malaria, snakebite, hemorrhoids, leprosy and jaundice^9^. It is also used to treat stomach ulcers and has antimicrobial, anti-leukemia and anti-tumor properties^10^. The polypeptide composition (a type of vegetable insulin) of Carla fruit and seed is very similar to insulin in terms of composition. The primary compounds that have hypoglycemic properties in this plant include quarantine, insulin-like peptides (plant insulin), cocorbutanoids, momordicin and oleanolic acid^11^.

One of the methods used for producing medicinal species is the use of hydroponic technology. The main advantages of hydroponic culture include increasing density per unit area, increasing crop production, the possibility of growing plants in all areas, reducing production stress, reducing problems caused by pests and diseases, saving water consumption and preserving water resources, the absence of invasive species, preserving food, control of environmental conditions, easy control of salt accumulation in root growth area, easier establishment of plants and no need for arrangement in crop rotation program^12^. Majid^13^ in a research on “evaluation of hydroponic and soil systems on lettuce performance” stated that most of the photosynthetic parameters were higher in hydroponic culture system. In general, the results of their research showed that performance of hydroponic system was significantly higher than that of the soil-based system. However, a significant decrease in dry matter content was observed under hydroponic conditions.

There are many nutritional formulas that have been the subject of many researches and studies for more than 60 years. One of the most famous type of these solutions, especially in research culture, is Hoagland’s solution. Mineral nutrients affect the growth and compounds of medicinal plants, and their mode of action is by affecting the enzymes involved in the biosynthesis of compounds^14^. It has been determined that under hydroponic conditions, nitrogen has positive effects on biomass, crop yield, chlorophyll and flavonoid levels, and essential oil quality^15,16^. Growth, the amount of flavonoids and essential oil and the antioxidant property of essential oil under hydroponic conditions are affected by the ratio and amount of mineral elements, especially phosphorus^16^. In a study, Sinkovic^17^ investigated the effects of different nutrient solutions (solutions enriched with nitrogen, potassium and phosphorus) on chicory plants under hydroponic conditions and reported that the highest contents of total phenol and total flavonoid were observed in nutrient solution enriched firstly with potassium, then nitrogen and finally phosphorus. Furthermore, the highest antioxidant potential was obtained in the nutrient solution enriched with nitrogen.

Silicon is the second element in the earth’s crust^18^ and is a constituent of plant tissues. Despite its abundance, silicon is never found in free form and is usually found in the form of oxides or silicates^18^. The major part of silicon in soil includes silicon presented in the solid phase, which is found in the structure of clay minerals and amorphous silicates. It is the most important primary source of silicon needed by plants, and is absorbed by plants in the form of acid anion^19^. Silicon absorption in plants is in the form of monosilicic acid and orthosilicic acid (H_2_SiO_4_) which are absorbed by diffusion, as well as the effect of root transpiration pressure through mass flow. After the absorption of silicon, the transfer of the element in the plant occurs through xylem and by the transpiration flow from the root to the shoot^20^. Silicon is an immobile element inside the plant which is finally deposited in the form of amorphous silicates (opal, silica gel or phytoliths) in all parts of higher plants such as cell walls, intercellular space, roots, leaves and reproductive organs, and becomes unavailable for the plant and will only have the role of strength and stability^21^. Based on the process of silicon absorption and transfer, which is active, passive and selective absorption, plants are classified as high, medium or non-accumulators of silicon^20^. Nowadays, the effects of silicon element in increasing resistance to pests and diseases, toxicity to heavy elements and increasing yield in many plant species have been proven^22^. The beneficial effects of silicon absorption vary from species to species^18^. Many plants have the ability of absorbing silicon, and depending on the species, the accumulated silicon content in biomass can range from 10 to more than 100 g per kg^23^. Silicon is the only element that does not harm plants through high accumulation in their tissues, although the role of silicon in plants is ignored due to its high abundance in the earth’s crust^24^. Silicon can reduce wheat lodging, reduce transpiration and increase the photosynthetic capacity of plants^25^. According to a report, silicon can increase the availability of phosphorus in soil and thus increase phosphorus absorption by plants. In fact, silicon increases the solubility of soil phosphorus by replacing the stabilized phosphorus between clays^26^. The consumption of silicon at the optimal level increases the tolerance of plants to salinity and drought and even increases the permeability of water in soil^27^. Silicon causes the uniform distribution of manganese in the leaves and increases the plant’s tolerance against manganese toxicity. The increase in calcium concentration due to the application of silicon increases membrane stability in plants under stress. Application of silicon can also lead to better storage of potassium under stress conditions, which reduces the adverse effect of drought stress^28^. Increase of growth and improvement of roots volume and weight by using optimal silicon nutrition can lead to an increase in the total surface of elements absorption. By reducing the amount of transpiration, silicon can maintain the transport of nutrients inside the plant as well as water sufficiency conditions^29^. In cut flower of rose, the treatment of silicon prevented the decomposition of chlorophyll and increased the life of the flower^30^. In strawberry, the use of silicon showed significant effects on the nutritional elements and quality of the fruit depending on the form of silicon, its concentration and application method. Based on their obtained results, application of silicon dioxide during the plant growth stage, especially in nano scale, caused a further increase in nutrients and improved the quality of strawberry fruit^31^. During a research conducted by Fekry^32^, it was shown that use of two selenium and silicon elements at a concentration of 50 mg L^− 1^ along with humic acid was the best treatment for improving yield, cluster weight, and physic-chemical characteristics of date fruit var. Barhi. Moreover, growth characteristics such as the amount of chlorophyll and nutrient status of the leaves increased by the use of these substances compared to the control.

This research was conducted to evaluate the effect of nutrient solution and the effect of foliar application of useful element silicon on growth, yield and some biochemical traits of Carla under hydroponic conditions.

## Materials and methods

### Location, plant planting method, growth conditions and application of treatments

This research was carried out in the laboratory and research greenhouse of the Medicinal Plants Department of Arak University at an altitude of 1743 m above sea level, geographical coordinates of 34.082.182° North latitude and 49.677295° East longitude, and during the fall and winter of 2021 and spring of 2022. The species studied in this research was the medicinal plant Carla. The current research was carried out in the form of a factorial experiment based on a complete randomized design with three replications as a hydroponic culture in pots under greenhouse conditions. The research treatments were designed as follows: the first factor included concentrations of nutrient solution at four levels of full strength Hoagland nutrient solution as control, double strength Hoagland, half strength Hoagland (½) and one-fourth Hoagland (¼), and the second factor included foliar spraying with sodium silicate (Source of silicon was Sodium silicate, manufacturer was Merck Co., Germany. Composition was Na_2_SiO_3_, MW = 122.06 g.mol^− 1^) at four levels (0, 50, 100 and 150 mg L^− 1^). Carla seeds were obtained from Zabol Natural Resources Research Center. First, the seeds were placed in a wet cloth for 24 h. Then, they were planted in culture tray containing coco peat and perlite, and normal water was used to keep the seeds moist until they germinated. At 4–6 leaf stage, the plants were transferred from the cultivation trays to the pots (diameter 23 cm and height 30 cm) containing coco peat-perlite in the ratio (2:1). After the transfer, feeding of the plants with different strengths of Hoagland’s solution, which was prepared according to Table 1, continued as a solution in irrigation water until the end of the growth period. Also, sodium silicate foliar spraying was performed at four levels once every 10 days as a manual spray on the leaves of the Carla. All the operations related to growing and caring of Carla plant in the greenhouse, such as twisting, connecting the main and secondary stems of the plant around the thread, were performed until the end of the plant’s growth season. During the growth period, the temperature was maintained at 24 °C ± 4 and relative humidity at 50%±5^33^.

**Table 1:** The concentration of salts used in nutrient solutions in the experiment.

Chemical compound	Nutrient solution compounds	Concentration
	Macroelements (mmol.L^− 1^)	
Potassium nitrate	KNO_3_	5
Calcium nitrate	NO_3_)_2_.4H_2_O) Ca	5
Magnesium sulphate	MgSO_4_.7H_2_O	2
Potassium dihydrogen phosphate	KH_2_PO_4_	1
	**Microelements (mg kg** ^**− 1**^ **)**	
Boric acid	H_3_BO_3_	2.86
Manganese chloride	MnCl_2_.4H_2_O	1.81
Zinc sulfate	ZnSo_4_.5H_2_O	0.22
Copper sulfate	CuSO_4_.5H_2_O	0.08
Molybdic acid	H_2_MoO_4_.H_2_O	0.02
Ferric-EDTA	Fe-EDTA	5

## Assessed traits

### Morphological, growth, and yield traits

**Fig. 1:**
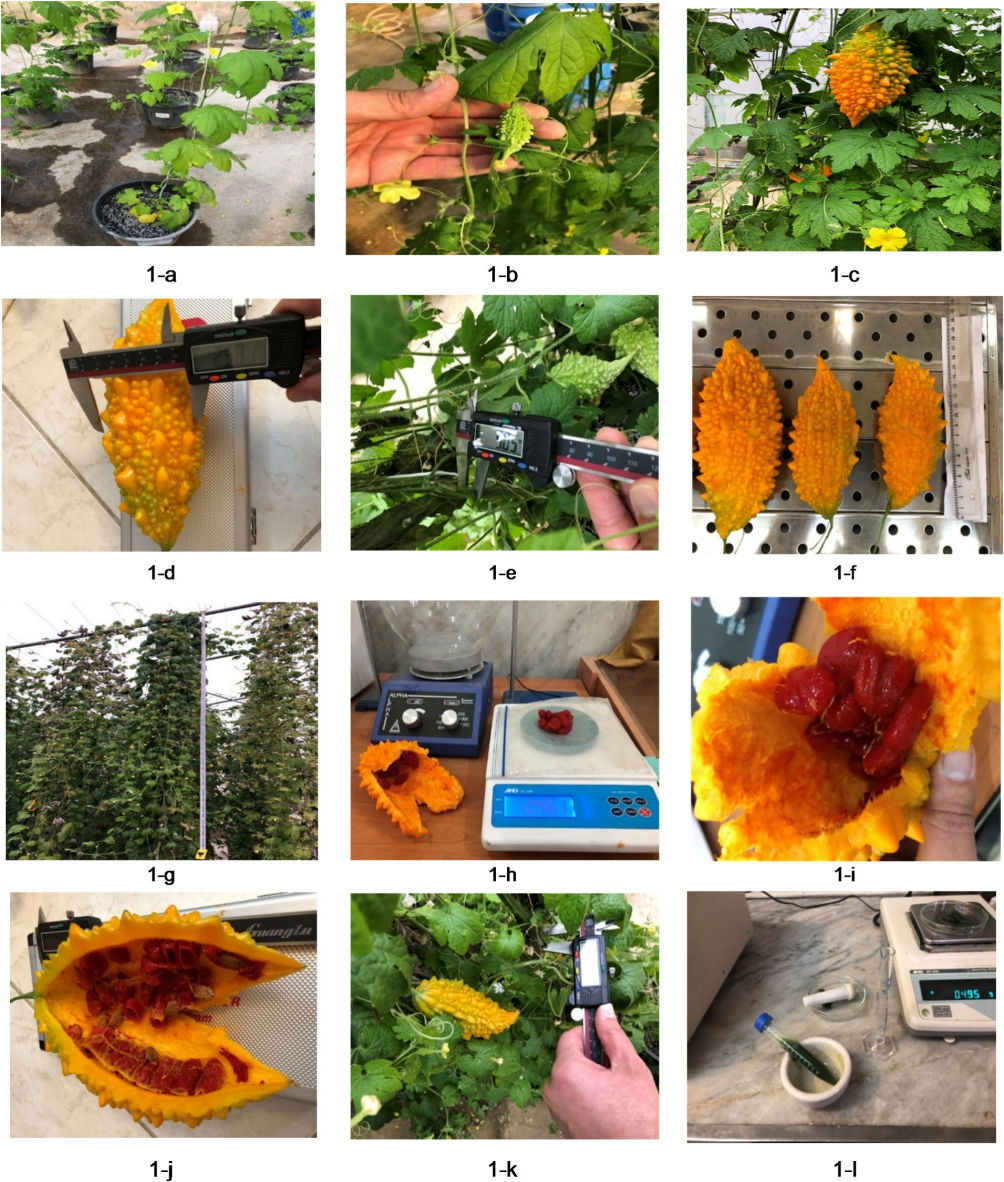
Some measured traits in Carla under the influence of different strengths of Hoagland’s solution and different levels of silicon.

In this research, the following morphological-functional traits were measured: flowering time, flowers number, fruits number, fruit diameter, fruit length, fruit fresh and dry weights, number of seeds per fruit, seed fresh and dry weights, main stem length (MSL), secondary stem length (SSL), main stem diameter (MSD), secondary stem diameter (SSD), nodes number, internodes length, fresh and dry weights of aerial parts, root fresh and dry weights, time of fruit harvesting (Fig. 1).

### Biochemical traits

The amounts of chlorophyll a, chlorophyll b and carotenoids were measured using the method of Lichtenthaler^34^. In this method, an amount of 0.1 g tissue was separated from fresh leaves and crushed and ground with 10 ml 80% acetone in a Chinese mortar. The obtained extracts were placed in a centrifuge at 10,000 rpm for 10 min, and 3 ml of the resulting solution was poured into the cuvette. The absorption value was read at three wavelengths of 663, 646 and 470 nm. In the present experiment, a model spectrophotometer device (speco 200 model spectrophotometer manufactured by Analyticjena, Germany) was used, and the following relationships were used for calculating the concentrations of chlorophyll a, chlorophyll b and carotenoids.

$$\:{\text{C}}_{\text{a}}=(19.3\times\:\text{A}0.086-663\times\:\text{A}647)\:\text{v}/100\text{w}$$

$$\:{\text{C}}_{\text{b}}=(19.3\times\:\text{A}647-3.6\times\:\text{A}663)\:\text{v}/100\text{w}$$

$$\:{\text{C}}_{\text{x}+\text{C}}=100\left(\text{A}470\right)-3.27\left(\text{C}\text{a}\right)-104\left(\text{m}\text{g}\:\text{C}\text{a}\right)/227$$

Where C_a_ is the amount of chlorophyll a, C_b_ is the amount of chlorophyll b, C_x+c_ is the total amount of carotenoids, V is the volume of the upper solution obtained from the centrifuge, W is the weight of the sample in g, and A is the light absorption in the respective wavelengths. The results of chlorophyll and carotenoid measurements were calculated and presented in terms of mg/g fresh weight (Fig. 1).

### Statistical analysis

Statistical calculations of the current research were performed using SAS software version 9.4 (https://support.sas.com/software/94/), and Duncan’s multi-range test was used to compare the means at 5% probability level. Tables and graphs were drawn using Excel software.

## Results and discussion

The results of analysis of variance (Table 2) showed that the simple effects of different strengths of Hoagland on all the morphological and functional traits, except for the ratio of fruits number to flowers number, were statistically significant at 1% probability level. Simple effects of different concentrations of silicon on the main and secondary stems diameters, nodes number, internode length, fresh and dry weights of the aerial part, yield of the aerial part, root fresh and dry weights, main and side shoots lengths, the ratio of the main shoots length and side shoots length, total biomass, flowering time and fruit harvesting time were statistically significant at 1% probability level. Furthermore, flowers number and fruits number were significantly affected at 5% probability level, and other traits were not significantly affected. The interaction effects of different strengths of Hoagland’s nutrient solution and silicon foliar application on the traits of internode length, ratio of main stem length to secondary stem length, ratio of fruit dry weight to biomass, flowering time and harvest time were significant at 1% probability level, and other traits were not significantly affected.

**Table 2:** The results of analysis of variance of the morphological-growth traits of Carla under the influences of different strengths of Hoagland’s nutrient solution and different silicon concentrations.

S.O.V	MS				
	HO	Si	HO*Si	Error	CV (%)
Df	3	3	9	32	
Flower number	4967.67 ^**^	324.34 ^*^	108.87 ^ns^	60.75	24.62
Fruit number	628.84 ^**^	53.04 ^*^	10.55 ^ns^	8.37	26.85
Fruit number/flower number	0.002 ^ns^	0.003 ^ns^	0.005 ^ns^	0.03	51.09
Fruit diameter (cm)	99.57 ^**^	1.70 ^ns^	0.67 ^ns^	0.87	4.31
Fruit length (cm)	48.52 ^**^	0.83 ^ns^	0.32 ^ns^	0.42	4.88
Fruit diameter /fruit length	0.0077 ^**^	0.00003 ^ns^	0.00003 ^ns^	0.00017	0.80
Fruit fresh weight (g)	9343.11^**^	160.25 ^ns^	63.25 ^ns^	82.52	8.30
Fruit dry weight (g)	3583.86 ^**^	61.47 ^ns^	24.26 ^ns^	31.65	14.35
Fruit dry weight/total biomass	0.116 ^**^	0.007 ^ns^	0.015 ^**^	0.0037	36.60
Seed number	100.882 ^**^	19.21 ^ns^	7.74 ^ns^	9.014	13.37
Seed fresh weight (g)	6911.96 ^**^	1185.40 ^ns^	467.91 ^ns^	610.46	9.18
Seed dry weight (g)	8815.57 ^**^	151.20 ^ns^	59.68 ^ns^	77.86	19.46
Main stem length (cm)	1653.38 ^**^	1407.63 ^**^	720.53 ^ns^	1238.20	11.57
Secondary stem length (cm)	20348.04 ^**^	1784.93 ^**^	89.40 ^ns^	154.91	12.63
MSL/SSL	2.15 ^**^	0.43 ^**^	0.30 ^**^	0.041	6.30
Main stem diameter (mm)	71.57 ^**^	4.82 ^**^	0.21 ^ns^	0.57	16.12
Secondary stem diameter (mm)	2.44 ^**^	0.19 ^**^	0.01 ^ns^	0.017	13.62
Nodes number	231.29 ^**^	185.83 ^**^	6.31 ^ns^	18.43	17.38
Internodes length (cm)	86.032 ^**^	11.61 ^**^	0.56 ^**^	0.028	1.57
Fresh weight of aerial parts (g)	1046.68 ^**^	8173.01 ^**^	4574.76 ^ns^	7648.08	10.24
Dry weight of aerial parts (g)	1118.21 ^**^	1014.34 ^**^	583.45 ^ns^	976.53	19.10
Aerial parts yield (g)	161202.64 ^**^	10082.45 ^**^	972.66 ^ns^	1434.14	20.42
Root fresh weight (g)	65984.29 ^**^	7515.71 ^**^	1445.38 ^ns^	833.29	4.24
Root dry weight (g)	8103.53 ^**^	1421.43 ^**^	62.07 ^ns^	67.11	5.24
Aerial parts weight/root weight	4.075 ^**^	0.157 ^ns^	0.033 ^ns^	0.059	21.85
Total biomass (g)	266576.11 ^**^	29657.28 ^**^	1357.15 ^ns^	2046.29	13.48
Flowering time (day)	4248 ^**^	402 ^**^	234 ^**^	0.90	5.77
Time of fruit harvesting (day)	5887 ^**^	621 ^**^	360 ^**^	2.25	6

Ho: strengths of Hoagland’s nutrient solution, Si: different concentrations of silicon, Ho*Si: the interaction effect of different strengths of Hoagland’s nutrient solution and different concentrations of silicon. **: significant at 1% level, *: significant at 5% level and ns: not significant.

According to means comparison of the data (Table 3), the highest flowers number (57.64), fruit number (20.07), fruit diameter (25.33 cm), fruit length (15.92 cm), fruit fresh weight (144.45 g), fruit dry weight (60.92 g), seed number (33.88) (Fig. 1-i and -j), seed fresh and dry weights (364.48 and 79.40 g, respectively) (Fig. 1-h), main and secondary stems lengths (437.64 and 145.24 cm, respectively), main and secondary stems diameters (7.52 and 1.49 mm, respectively), nodes number (40.49), fresh and dry weights of the aerial parts (1193.31 and 271.64 g, respectively), yield of aerial part (320.71 g), root fresh and dry weights (770.48 and 180.45 g, respectively), ratio of aerial part yield to root weight (1.78) and total biomass (499.54 g) were obtained in double strength Hoagland’s solution; these traits showed reduction when the strength of Hoagland’s solution decreased, so that the lowest values of these traits were observed at ¼ strength Hoagland’s solution. Also, according to the data in Table 3, the shortest flowering time, the shortest fruit harvesting time (the fastest time to harvest), the shortest node length and the lowest ratio of fruit diameter to fruit length (Fig. 1-d and -f) were observed at double strength Hoagland’s solution, and the highest values of these traits were observed when using ¼ strength Hoagland’s solution.

**Table 3:** The results of means comparison of morphological-growth traits in under the influence of different strengths of Hoagland’s nutrient solution.

Treatments	Hoagland Strength			
	1	2	½	¼
Flower number (No./plant)	37.81 ^b^	57.64 ^a^	18.01 ^c^	13.14 ^c^
Fruit number (No./plant)	12.81 ^b^	20.07 ^a^	6.18 ^c^	4.03 ^c^
Fruit diameter (cm)	22.73 ^b^	25.32 ^a^	19.39 ^c^	19.39 ^c^
Fruit length (cm)	14.10 ^b^	15.92 ^a^	11.78 ^c^	11.78 ^c^
Fruit diameter /fruit length	1.60 ^b^	1.59 ^b^	1.64 ^a^	1.64 ^a^
Fruit fresh weight (g/fruit)	119.22 ^b^	144.45 ^a^	86.92 ^c^	86.11 ^c^
Fruit dry weight (g/fruit)	45.30 ^b^	60.92 ^a^	25.29 ^c^	25.03 ^c^
Fruit dry weight/total biomass	0.116 ^c^	0.209 ^b^	0.060 ^d^	0.283 ^a^
Seed number (No./plant)	25.77 ^b^	33.88 ^a^	15.06 ^c^	15.06 ^c^
Seed fresh weight (g/plant)	295.87 ^b^	364. 48 ^a^	207. 11 ^c^	208. 01 ^c^
Seed dry weight (g/plant)	54.89 ^b^	79.40 ^a^	23.18 ^c^	23.51 ^c^
Main stem length (cm)	379.96 ^b^	437.64 ^a^	268.32 ^c^	161.66 ^d^
Secondary stem length (cm)	114.27 ^b^	145.24 ^a^	86.01 ^c^	48.53 ^d^
MSL/SSL	3.28 ^b^	2.98 ^c^	2.82 ^c^	3.78 ^a^
Main stem diameter (mm)	5.59 ^b^	7.52 ^a^	3.94 ^c^	1.77 ^d^
Secondary stem diameter (mm)	1.14 ^b^	1.49 ^a^	0.84 ^c^	0.43 ^d^
Nodes number (No./plant)	30.15 ^b^	40.49 ^a^	20.10 ^c^	8.03 ^d^
Internodes length (cm)	9.60 ^b^	119.31 ^a^	764.55 ^c^	497.23 ^d^
Fresh weight of aerial parts (g/plant)	959.79 ^b^	1193.31 ^a^	764.55 ^c^	497.23 ^d^
Dry weight of aerial parts (g/plant)	205.59 ^b^	271.64 ^a^	128.93 ^c^	48.21 ^d^
Aerial parts yield (g/plant)	229.09 ^b^	320.71 ^a^	141.17 ^c^	50.76 ^d^
Root fresh weight (g/plant)	708.31 ^b^	770.48 ^a^	636.31 ^c^	605.17 ^d^
Root dry weight (g/plant)	172.88 ^b^	180.45 ^a^	147.86 ^c^	123.19 ^d^
Aerial parts weight/root weight	1.31 ^b^	1.78 ^a^	0.95 ^c^	0.40 ^d^
Total biomass (g/plant)	400.11 ^b^	499.54 ^a^	289.73 ^c^	152.57 ^d^
Flowering time (day after planting)	54.6 ^c^	43.2 ^d^	79.9 ^b^	85.5 ^a^
Time of fruit harvesting (DAP)	69.6 ^c^	53.1 ^d^	63.6 ^b^	99.6 ^a^

Mean values with different letters in a column are significantly different at 5% level probability according to Duncan’s test (*p* < 0.05).

According to the data in Table 4, it was found that the traits of flower number (Fig. 1-a), fruit number (Fig. 1-b and -c), main and secondary stems lengths (Fig. 1-g), main and secondary stems diameters (Fig. 1-e and -k), node number, fresh and dry weights of aerial part, yield of aerial part, root fresh and dry weights, ratio of aerial part yield to root weight and total biomass had the highest values when sodium silicate applied at 100 mg L^− 1^, and the lowest values of these traits were seen in the control. Moreover, the shortest flowering time, the shortest fruit harvesting time (the fastest time to harvest) and the longest internode length were observed at the concentration of 100 mg L^− 1^ sodium silicate, and the lowest values of these traits were obtained in the control.

**Table 4:** The results of means comparison of morphological-growth traits in Carla the influence of different concentrations of silicon.

Treatments	Silicon concentrations (mg kg^− 1^)			
	0	50	100	150
Flowers number (No./plant)	26.87 ^c^	27.97 ^bc^	37.99 ^a^	33.76 ^ab^
Fruits number (No./plant)	8.51 ^c^	9.62 ^bc^	13.18 ^a^	11.79 ^ab^
Main stem length (cm)	260.21 ^c^	294.41 ^b^	337.80 ^a^	321.17 ^ab^
Secondary stem length (cm)	83.03 ^c^	94.04 ^b^	110.70 ^a^	105.29 ^ab^
MSL/SSL	3.34 ^a^	3.39 ^a^	2.98 ^c^	3.15 ^b^
Main stem diameter (mm)	3.90 ^c^	4.55 ^b^	5.37 ^a^	4.99 ^ab^
Secondary stem diameter (mm)	0.81 ^c^	0.94 ^b^	1.10 ^a^	1.05 ^ab^
Nodes number (No./plant)	20.05 ^b^	23.01 ^b^	28.86 ^a^	26.85 ^a^
Internodes length (cm)	11.85 ^a^	11.06 ^b^	9.59 ^d^	10.23 ^c^
Fresh weight of aerial parts (g/plant)	748.64 ^c^	829.94 ^b^	829.29 ^b^	902.01 ^a^
Dry weight of aerial parts (g/plant)	129.61 ^d^	150.88 ^c^	194.99 ^a^	178.89 ^b^
Aerial parts yield (g/plant)	154.28 ^c^	169.20 ^bc^	218.17 ^a^	200.09 ^ab^
Root fresh weight (g/plant)	664.35 ^b^	681.24 ^a^	695.88 ^a^	698.81 ^a^
Root dry weight (g/plant)	124.71 ^c^	151.99 ^b^	166.63 ^a^	163.05 ^a^
Aerial parts weight/root weight	1.14 ^ab^	1.26 ^a^	1.03 ^b^	1.02 ^b^
Total biomass (g/plant)	362.66 ^a^	384.64 ^a^	323.29 ^b^	271.37 ^c^
Flowering time (day after planting)	60.9 ^c^	55.2 ^d^	67.2 ^b^	72.9 ^a^
Time of fruit harvesting (DAP)	81.9 ^a^	70.2 ^b^	81.9 ^a^	81.9 ^a^

Mean values with different letters in a column are significantly different at 5% level probability according to Duncan’s test (*p* < 0.05).

The results showed that increasing the strength of Hoagland’s solution had a positive effect on morphological traits. This effect was caused by the difference in the concentrations of the elements, which increased the amount of elements such as NPK as the concentration increased, leading to the highest rate of absorption by the plant as well as an increase in morphological traits. The reason for the positive response of the Carla plant to the high concentration of the Hoagland solution is the plant’s high demand for nutrients and the growing season. In winter, due to low light and temperature, evaporation and transpiration in the plant decrease, resulting in reduced nutrient solution absorption. To compensate for the reduced absorption caused by the decrease in evaporation and transpiration, producers increase the concentration of the nutrient solution in hydroponic systems. Therefore, in the present experiment, the likely reduction in nutrient solution absorption during winter may have led to the highest concentration of the Hoagland solution yielding better results for this plant compared to other concentrations. Based on the studies conducted by Kang^35^, use of different concentrations of nutrient solution in sage plant had a significant effect on morphological traits such as dry weights of roots, shoots and the whole plant. It was also reported in another research that different concentrations of Hoagland on physalis fruit improved its morphological traits^36^. In other reports, researchers stated that different strengths of Hoagland’s solution resulted in an increase in the essential oil of peppermint and improved the morphological and phytochemical characteristics of costmary^37^.

Furthermore, increasing the applied concentration of silicon enhanced the amount of morphological traits in the present study. Although silicon is not an essential element in plant nutrition, it has been proven to be useful for the growth and development of many plant species, so that numerous studies have shown its positive effects on plant growth and performance^38^. Gong^39^ concluded that application of silicon improved water status of the plant, which led to an increase in fresh and dry weights of the wheat plant. In a research that investigated the effect of silicon on salinity resistance of zucchini (*Cucurbita pepo* L. cv. Rival) in hydroponic culture, 1 mM silicon increased vegetative growth, fruit yield, plant fresh and dry weights, and plant photosynthesis rate, and eliminated the negative effect of salinity^40^. The results of this research are consistent with the current research.

**Fig. 2:**
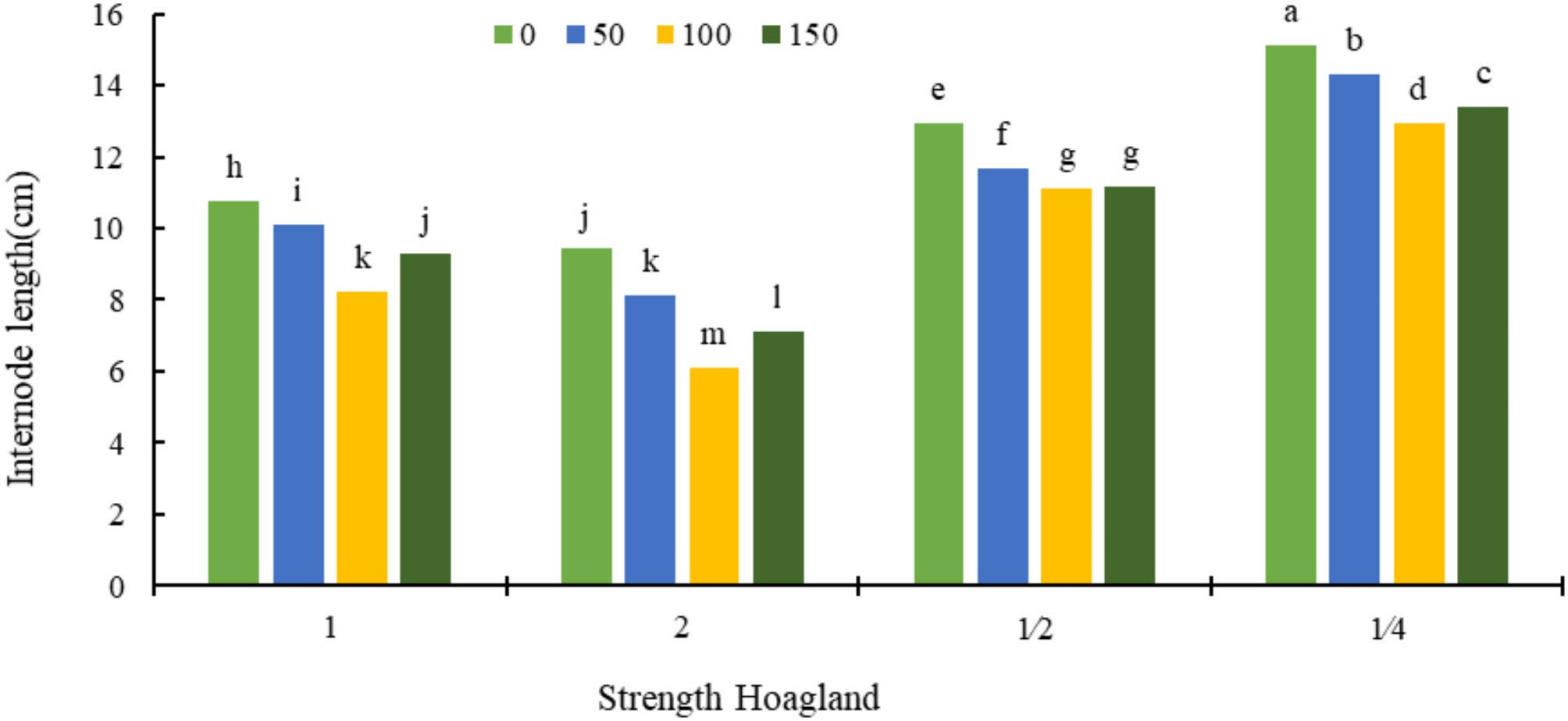
The interaction effect of Hoagland’s different strengths and different silicon concentrations (mg kg^− 1^) on internode length. Different letters indicate significant differences among the means at 5% level based on Duncan’s test.

Based on the data of Fig. 2, the shortest internode length was observed in the combined treatment of double strength Hoagland’s solution and 100 mg L^− 1^ sodium silicate (6.1 cm) and the highest value was observed when the strength of Hoagland solution was ¼ without silicon application.

In the present study, the increase of nutrients and silicon concentrations brought about a reduction in internode length and production of stronger stem. Application of silicon in plant increases the absorption of carbon, nitrogen and phosphorus, leading to the improvement of agronomical traits^41^. It is possible that silicon plays an important role in the strength and size of wall pores, as well as the diameter and length growth of cells, especially the xylem of plants, by forming complexes with wall compounds^42^. In an investigation into the effect of silicon on the quantitative and qualitative characteristics of early and semi-late varieties of sugarcane, it was reported that application of silicon in the form of ortho-silicic acid (OSA) had favorable effects on the yield characteristics of sugarcane, including sugarcane diameter, internode length, stem height and increased these traits^43^.

**Fig. 3:**
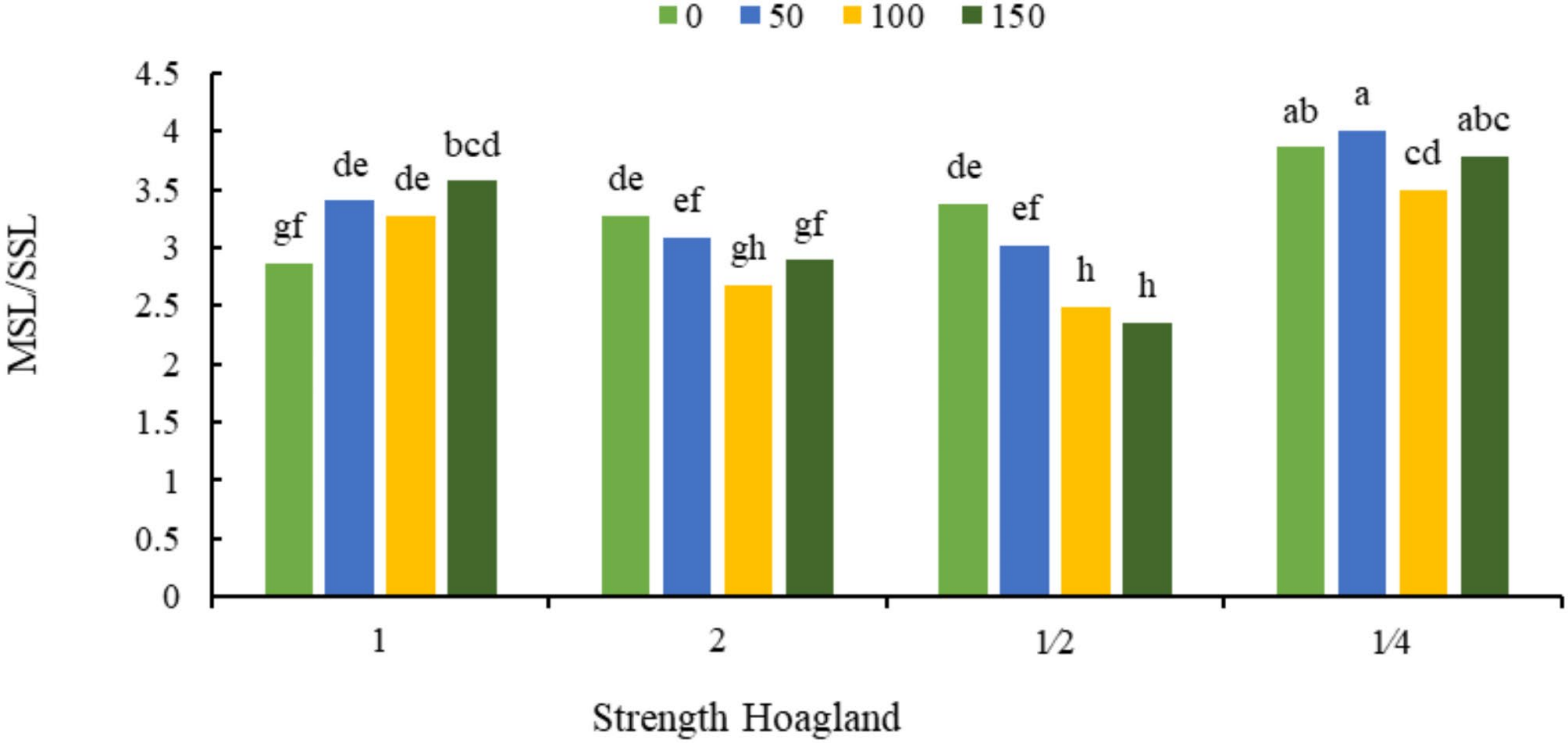
The interaction effect of Hoagland’s different strengths and different silicon concentrations (mg kg^− 1^) on the ratio of main stem length to secondary stem length (MSL/SSL). Different letters indicate significant differences among the means at 5% level based on Duncan’s test.

According to Fig. 3, the highest ratio of the main stem length to the secondary stem length was related to 50 mg L^−^ sodium silicate foliar spraying and the application of ¼ strength Hoagland (4.01), and the lowest value of this trait was related to the application of 150 mg L^− 1^ of sodium silicate and half strength Hoagland (2.35).

In the current study, the reduction of nutrients caused a decrease in the growth of the secondary stem and an increase in the longitudinal growth of the main stem, and the application of silicon reduced this effect. The increase in the ratio of the main stem length to the secondary stem length stemmed from the imbalance of nutrients in the plant, which can be alleviated by the application of silicon^44^.

**Fig. 4:**
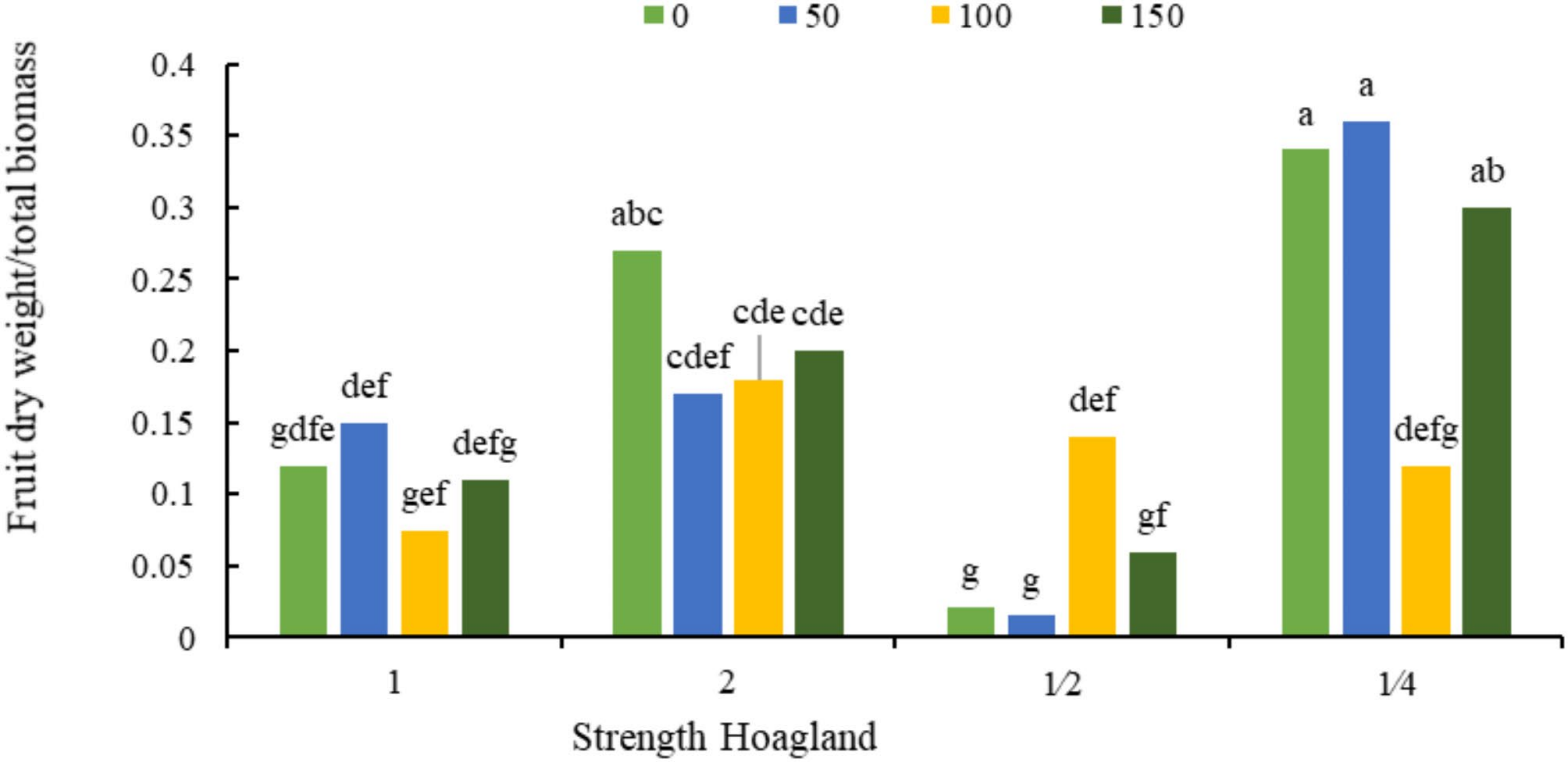
The interaction effect of Hoagland’s nutrient solution strengths and different silicon concentrations (mg/L) on the ratio of fruit dry weight to total biomass. Different letters indicate significant differences among the means at 5% level based on Duncan’s test.

According to Fig. 4, the highest ratio of fruit dry weight to biomass weight was obtained from combined treatment of 50 mg/L sodium silicate and ¼ strength Hoagland’s solution (0.36), and the lowest value of this trait was observed in the treatment of 150 mg/L sodium silicate and half strength Hoagland’s solution (0.015).

In the current study, reduction of nutrient caused the plant to decrease its biological growth and increase fruit growth and seed production, and silicon application reduced this effect. The increase in the ratio of fruit dry weight to biomass is caused by the imbalance of nutrients in the plant, which is balanced by the application of silicon^44^. Application of silicon increased the efficiency of nutrients N, P and K and improved plant performance^29^. It has been reported that application of amorphous silicon increased wheat yield and soil carbon absorption^45^.

**Fig. 5:**
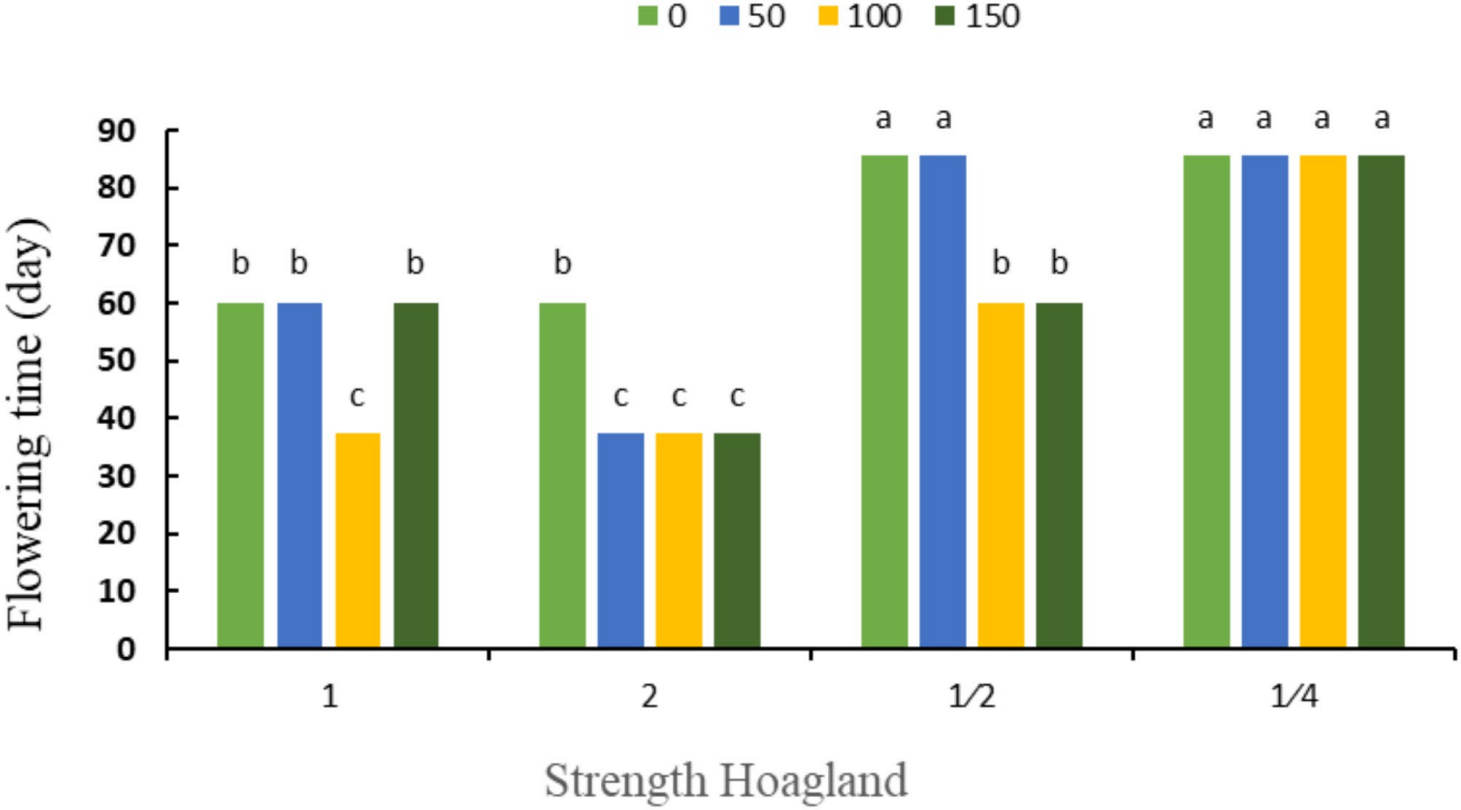
The interaction effect of Hoagland’s nutrient solution strengths and different concentrations of silicon (mg kg^− 1^) on flowering time. Different letters indicate significant differences among the means at 5% level based on Duncan’s test.

According to Fig. 5, the earliest flowering time was observed in double strength Hoagland’s solution and foliar spraying of 50, 100 and 150 mg L^− 1^ sodium silicate, and the latest flowering time was seen in ¼ strength Hoagland’s solution, showing that it was not affected by the use of silicon.

**Fig. 6:**
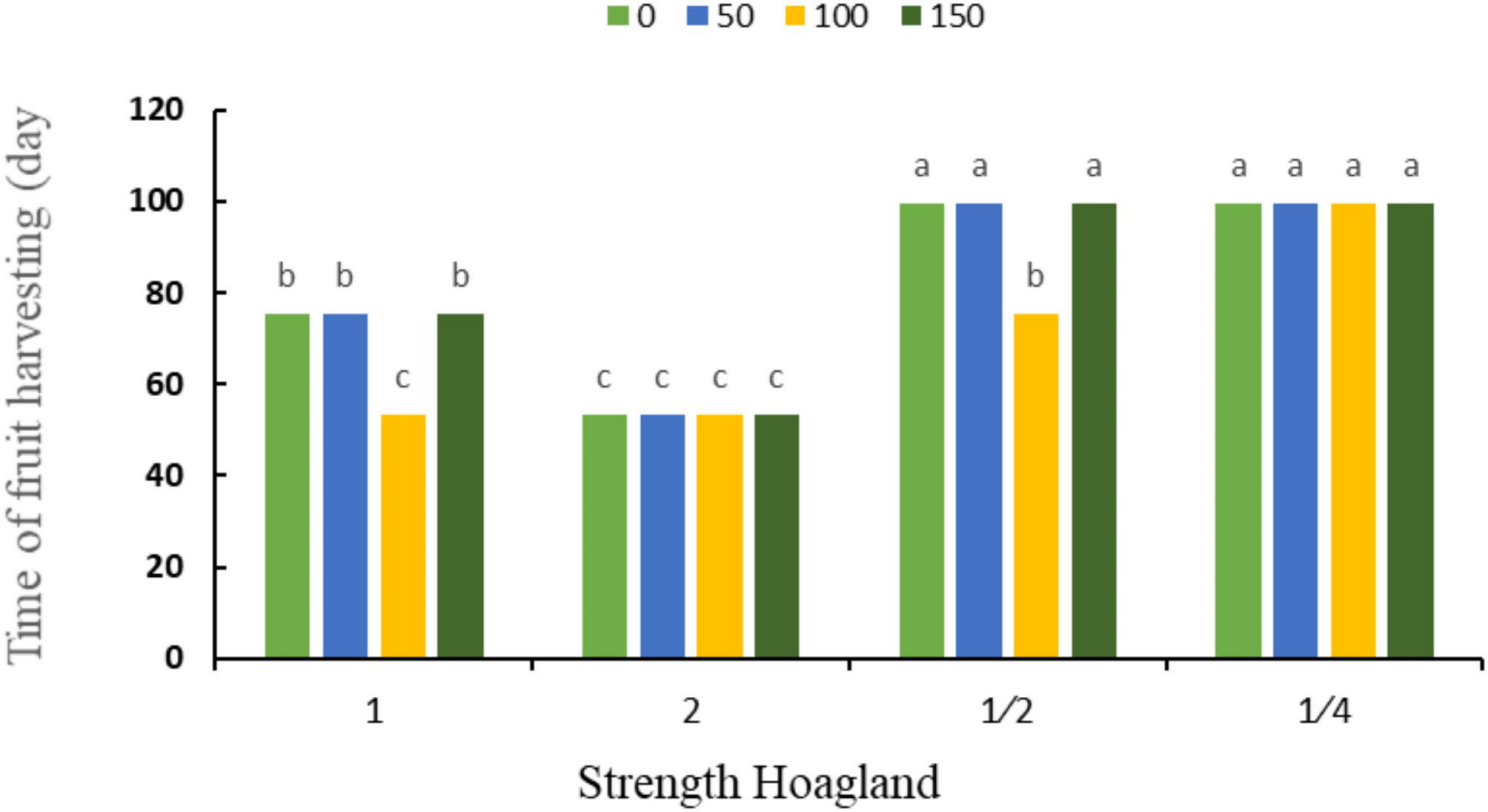
The interaction effect of Hoagland’s nutrient solution strengths and different concentrations of silicon (mg kg^− 1^) on fruit harvesting time. Different letters indicate significant differences among the means at 5% level based on Duncan’s test.

Moreover, according to Fig. 6, the shortest harvest time was observed in double strength Hoagland and foliar spraying of 50, 100 and 150 mg L^− 1^ sodium silicate, and the longest harvesting time was seen in ¼ strength Hoagland’s solution, regardless of silicon application.

A balanced supply of nutrients is very important for optimal yield and fruit quality^46^. Phosphorus is an essential component of nucleic acids, phospholipids and energy-rich phosphate compounds, so it plays an important role in the growth of different parts of plant and resistance against disease. Phosphorus deficiency can stop plant growth and reduce yield and quality^47^. Many papers introduced silicon (Si) as a beneficial plant nutrient which stimulates the growth and yield of various crops^48,49^. Silicon affects endogenous plant hormones including jasmonic acid and salicylic acid, and causes changes in gene expression and consequently biochemical changes such as antioxidant enzymes activity, chlorophyll content, polyamine content, soluble protein content, water potential and Rubisco content^50^. Therefore, it can be said that silicon caused early flowering time and early harvest time by balancing nutrient and affecting endogenous hormones.

According to the results of variance analysis (Table 5), the simple effect of different strengths of Hoagland’s solution and different levels of silicon on biochemical traits such as chlorophyll a, chlorophyll b and carotenoids (Fig. 1-l) was significant at 1% probability level. In addition, the interaction effects of Hoagland’s solution strength and silicon concentrations on the amount of chlorophyll a were significant at % probability level, but on the amount of chlorophyll b and carotenoids were not significant.

**Table 5:** The results of analysis of variance related to the physiological traits of Carla under the influence of different strengths of Hoagland’s nutrient solution and different silicon concentrations.

S.O.V	MS				
	HO	Si	HO*Si	Error	CV (%)
Df	3	3	9	32	
Chlorophyll a	5.54 ^**^	1.01 ^**^	0.39 ^**^	0.039	3.64
Chlorophyll b	6.35 ^**^	0.83 ^**^	0.11 ^**^	0.068	21.6
Carotenoids	0.33 ^**^	0.034 ^**^	0.0096 ^ns^	0.0077	5.31

Ho: strengths of Hoagland’s nutrient solution, Si: different concentrations of silicon, Ho*Si: the interaction effect of different strengths of Hoagland’s nutrient solution and different concentrations of silicon. **: significant at 1% level, *: significant at 5% level and ns: not significant.

The results of means comparison (Table 6) showed that the highest amounts of chlorophyll a and b were found in double strength Hoagland’s solution (1.95 and 0.63 mg/g, respectively) and the lowest amounts were observed in ¼ strength Hoagland’s solution (1.49 and 0.12 mg/g, respectively). Also, the amount of carotenoids was the highest in ¼ strength Hoagland’s solution (0.58 mg/g) and was the lowest in double strength Hoagland’s solution (0.47 mg/g). Moreover, the results of means comparison showed that the highest effects of different silicon concentrations on chlorophyll a and b traits were obtained at concentration of 150 mg L^− 1^ sodium silicate (1.80 and 0.45 mg/g, respectively) and the lowest values were obtained in the control. Moreover, sodium silicate control treatment (0 mg L^− 1^) contained the highest amount of carotenoids (0.54 mg/g) and concentration of 50 mg L^− 1^ had the lowest amount (0.50 mg L^− 1^).

**Table 6:** The results of means comparison related to physiological traits of Carla under the influence of different strengths of Hoagland’s nutrient solution and different silicon concentrations.

Treatments	Hoagland Strength			
	1	2	1/2	1/4
Chlorophyll a (mg/g FW)	1.82 ^b^	1.95 ^a^	1.56 ^c^	1.49 ^b^
Chlorophyll b (mg/g FW)	0.51 ^b^	0.63 ^a^	0.27 ^c^	0.12 ^d^
Carotenoids (mg/g FW)	0.49 ^c^	0.47 ^c^	0.55 ^b^	0.58 ^a^

		Silicon concentrations (mg kg^− 1^)		
	0	50	100	150
Chlorophyll a (mg/g FW)	1.63 ^c^	1. 64 ^c^	1.77 ^b^	1.80 ^a^
Chlorophyll b (mg/g FW)	0.27 ^c^	0.37 ^b^	0.44 ^a^	0.45 ^a^
Carotenoids (mg/g FW)	0.54 ^a^	0.50 ^b^	0.53 ^ab^	0.52 ^b^

Mean values with different letters in a column are significantly different at 5% level probability according to Duncan’s test (*p* < 0.05).

The reason for the increase of photosynthetic pigments in double strength Hoagland’s solution can be related to the higher concentration of nitrogen because the increase of photosynthesis and chlorophyll content is directly affected by nitrogen. Therefore, when nitrogen is sufficient during the process, the amount of photosynthetic pigments increases^51^. Also, the results obtained in this research indicate the positive effect of silicon on chlorophyll content. In a study by Asmar^52^, they reported that chlorophyll content of banana seedlings increased in sodium silicate treatment. It was also reported in another study that application of silicon had a positive effect on the chlorophyll content of soybean seedlings in hydroponic culture^53^. The results of these researches are consistent with the results of the present study.

**Fig. 7:**
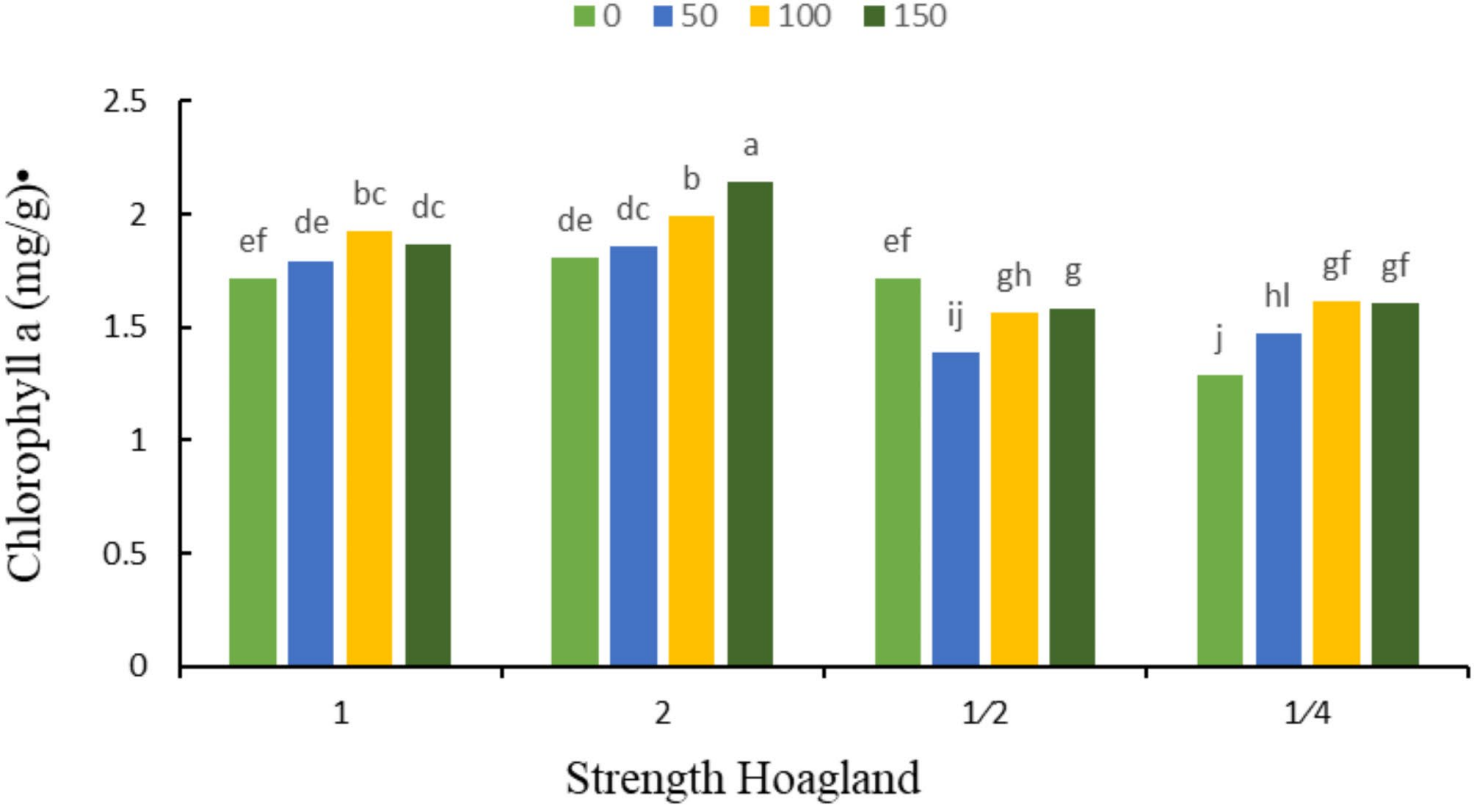
The interaction effect of different strengths of Hoagland’s solution and different concentrations of silicon (mg kg^− 1^) on chlorophyll a. Different letters indicate significant differences among the means at 5% level based on Duncan’s test.

According to data in Fig. 7, the highest amount of chlorophyll a was observed in double strength Hoagland’s solution and foliar spraying of 150 mg L^− 1^ sodium silicate, and the lowest amount of chlorophyll a was related to ¼ strength Hoagland’s solution without no silicon application.

The effects of silicon vary among plant species, but generally, it increases the efficiency of water use and photosynthesis, as well as correct nutrient imbalances^54,44^. Supplying silicon to cucumber plants had a positive effect on photosynthesis rate and chlorophyll fluorescence. Also, the contents of chlorophyll a, chlorophyll b, total chlorophyll and carotenoids increased^55^. It seems that silicon increased the amount of leaf chlorophyll with a protective effect on photosynthetic pigments through increasing the antioxidant activity of the leaf or stimulating the reactions that lead to the production of these pigments or a combination of both paths^56^;^53^).

## Conclusion

The results of the present study showed that double strength Hoagland’s solution had a better effect on all morphological traits than other concentrations, Furthermore, this strength decreased the times of flowering and fruit harvesting. On the other hand, besides reducing the times of flowering and fruit harvesting, concentrations of 100 and 150 mg L^− 1^ sodium silicate proved to be more effective on the measured traits. The interaction effects of silicon and different strengths of Hoagland were significant on flowering time, fruit harvesting time and chlorophyll a, but were not significant on other traits.

## Data Availability

The data presented in this study are available on request from the corresponding authors.
